# Radiation-Induced Sarcoma of the Head and Neck: A Review of the Literature

**DOI:** 10.3389/fonc.2018.00449

**Published:** 2018-10-17

**Authors:** Lorenzo Giannini, Fabiola Incandela, Marco Fiore, Alessandro Gronchi, Silvia Stacchiotti, Claudia Sangalli, Cesare Piazza

**Affiliations:** ^1^Department of Otorhinolaryngology, Maxillofacial and Thyroid Surgery, Fondazione IRCCS National Cancer Institute of Milan, University of Milan, Milan, Italy; ^2^Department of Otorhinolaryngology–Head and Neck Surgery, ASST Santi Paolo e Carlo, University of Milan, Milan, Italy; ^3^Department of Surgery, Sarcoma Unit, Fondazione IRCCS National Cancer Institute of Milan, Milan, Italy; ^4^Adult Mesenchymal and Rare Tumor Medical Oncology Unit, Department of Medicine, Fondazione IRCCS National Cancer Institute of Milan, University of Milan, Milan, Italy; ^5^Department of Radiotherapy, Fondazione IRCCS National Cancer Institute of Milan, University of Milan, Milan, Italy

**Keywords:** sarcoma, head and neck, radiotherapy, radiation-induced, surgical treatment

## Abstract

In the last decades, radiotherapy (RT) has become one of the cornerstones in the treatment of head and neck (HN) malignancies and has paralleled an increase in long-term patient survival. This lead to a concomitant increase in the incidence of radiation-induced sarcomas (RIS) of the irradiated field, with an annual rate up to 0.17%. The new techniques of irradiation do not seem to influence the risk of RIS of the HN (RISHN), which mainly develop within the middle-dose field. The median latency of RISHN after RT is 10–12 years and osteosarcoma is the most represented histotype, even though there is a high variability in time of occurrence and histological features observed. There is no clear evidence of predisposing factors for RISHN, and genetic findings so far have not revealed any common mutation. Early clinical diagnosis of RISHN is challenging, since it usually occurs within fibrotic and hardened tissues, while radiological findings are not pathognomonic and able to differentiate them from other neoplastic entities. Given the highly aggressive behavior of RISHN and its poor sensitivity to chemotherapy, radical surgery is the most important prognostic factor and the only curative option at present. Nevertheless, the anatomy of the HN district and the infiltrative nature of RIS do not always allow radical intervention. Therefore, a wise integration with systemic therapy and, when feasible, re-irradiation should be performed. Future findings in the genomic features of RISHN will be crucial to identify a possible sensitivity to specific drugs in order to optimize a multimodal treatment that will be ideally complementary to surgery and re-irradiation.

## Introduction

The long-term carcinogenic potential of ionizing radiations is well known and has been described since 1902 (1). In the last decades an increased number of radiation-induced (RI) neoplasia have been observed, due to—among other reasons—a substantial improvement of patients survival (2). The commonest histological subtypes of RI tumors are squamous cell carcinoma (SCC) followed by sarcoma, even though many types of cancers have been possibly described after radiotherapy (RT) (3).

RI sarcomas of the head and neck (RISHN) are very rare entities, and characterized by poor long-term outcomes. Their incidence is quite low and variable: among the largest retrospective studies available in the literature, the reported annual incidence is 0.06–0.17% (4), compared to a 1.6% incidence when all body RIS are considered (5). As a matter of fact, even a retrospective study performed at the National Cancer Institute of Milan, Italy, the leading center for treatment of sarcoma in our country, found only 5 cases of possible RISHN among 206 SHN treated between 1990 and 2010 (6).

The median time of latency after RT is reported to be 10–12 years, while the arbitrary cut-off used to distinguish RIS from sporadic sarcomas is 3–4 years after RT. However, it seems that the time of occurrence of cutaneous angiosarcoma, for example, may be shorter than for other histotypes (4, 6–8). Moreover, a recent multicenter study showed how the association of chemotherapy may significantly shorten the latency of the RI-related second tumor occurrence (8).

There is no evidence of a specific site in the HN in which RISs preferentially occur, even though different Chinese studies indicate the paranasal sinuses as the most commonly affected region (4, 9). However, this seems to be a bias due to the high prevalence of nasopharyngeal cancer in the Eastern population, with the nasopharynx and paranasal sinuses being the most irradiated subsites in such an epidemiological scenario.

In the current literature there is a substantial paucity of updated and comprehensive reviews on RISHNs. Moreover, many reports tend to analyze only clinical records from their own center, nearly always considering patients treated in a long time span, even several decades before, by obsolete treatments. In this way, there is a concrete risk to apply outdated data and concepts to the modern scenario, characterized by completely different RT techniques and therapeutic algorithms.

The aim of the present review is therefore to offer an up-to-date summary on this topic, with special emphasis to the ultimate molecular findings, RT technologies, and treatment possibilities of such a devastating disease.

## Radiation and carcinogenesis

Even though the effects of radiations in humans have been described since the beginning of the twentieth century, only the incidence and mortality for solid cancers and leukemia among nuclear bomb survivors in Japan provided us with the basis for understanding the functional dose-response relationships (10). Firstly, there is no “safety threshold” below which second malignancies may not occur, although a consistent number of dosimetric studies found that RI tumors occur close to the primary field of RT (within 5 cm) in a variable rate of 43–90% of patients (10). This can be explained by the main outcome of RT within the high-dose region, represented by the cell-killing effect, while the cellular repopulation in the surrounding areas receiving a lower dose would favor the clonal maintenance of mutated lines (10). It has been suggested that the highest risk of RI malignancies may be observed at sublethal cellular doses (6); nevertheless, a reduction in risk at high doses has not been shown, except for thyroid cancer (11).

Another issue on which the literature has recently focused its attention is the use of new RT techniques, with associated long-term risks. Intensity modulated radiation therapy (IMRT) and volumetric modulated arc therapy (VMAT) are nowadays the most common methods of RT used to treat HN tumors. These techniques consist in an evolution of the three-dimensional conformal RT (3D-CRT), in which it is possible to refine the high-dose conformity around cancers with complex shapes, in order to reduce the dose received by surrounding organs (12). This allows irradiating the target tissue with higher doses, while simultaneously reducing the dose delivered to areas close to the lesion. From a functional point of view, these techniques ensure less acute toxicity than traditional RT while, on the other hand, the low-dose irradiation fields become wider (12, 13). The worries about such techniques are based on the aforementioned principle of a relatively high impact of low-dose irradiation, in contrast with the high rate of cell death observed in high-dose areas. In fact, some authors have argued that IMRT/VMAT are likely to almost double the incidence of second malignancies compared with conventional RT techniques from about 1 to 1.75% for patients surviving 10 years (14). However recent studies including large series of patients did not find significant differences between IMRT, VMAT, and 3D-CRT in terms of second primary cancer risk (15, 16). Indeed, it cannot be ruled out that the potentially damaging consequences of the larger low-dose component surrounding the target in IMRT/VMAT are balanced by the higher cancerogenic effects observed in a smaller area receiving an intermediate-dose in 3D-CRT (12).

Also intensity modulated proton therapy (IMPT) is a promising approach to reduce commonly seen post-RT acute and chronic adverse effects (13). However, costs of IMPT greatly exceed those of IMRT and world-wide capacity of implementation of such a technique is, so far, rather limited. As a consequence, knowledge of IMPT effects on RISHN are still scant and far to be quantitatively meaningful.

Even the role of carbon ion radiotherapy is now under investigation, especially for management of recurrent HN cancer and RIS. This technique produces a higher linear energy transfer with greater relative biological effectiveness compared to photon and proton beams (14). However, even in this field, before seeing a substantial change in RIS epidemiology we will probably need some decades from now.

## Genomic correlations

The mechanisms involved in the cancerogenesis of RIS are represented by damage to double-stranded DNA, resulting in genomic instability (17). Many studies have looked for mutations implicated in the etiology of RIS: for example, it has been demonstrated the role of cMyc amplification in RI angiosarcomas, which is not found in other types of RIS (18).

Other authors have investigated the role of the tumor suppressor retinoblastoma 1 (RB1) levels in osteoblasts in the occurrence of RI osteosarcoma (RIOS) (19). In fact, RB1 activation induces the expression of a panel of proteins called senescence-associated secretory phenotypes (SASP), that is formed by cytokines (with interleukine-6 the most important) and complement proteins (19). During RT, the RB1-SASP pathway initiates a process of cellular senescence, resulting in immunologic recognition of damaged cells by natural killer T cells. A lack of this oncosuppressive system, due to the mutagenic potential of RT, may favor the occurrence of RIS (19).

Moreover, Hadj-Hamou et al. performed a comparative analysis in transcriptome modification between RIS and sporadic sarcomas (20). It was found that RIS are characterized by mitochondrial dysfunction that may be at the origin of a chronic endogenous oxidative stress: it is likely that such a phenomenon causes the alterations in pathways that lead to RIS (20).

For what concerns the HN region, recent studies examined the change in expression of p53 and one of its regulator proteins Murine Double Minute 2 (Mdm2), comparing their levels in *de novo* and in RIS (21). It was seen that p53 was overexpressed in RIS, while Mdm2 amplification was more represented in *de novo* tumors (21). Moreover, Mdm2-p53 interaction has gained interest in the last years, since a better response to chemotherapy was found in patients Mdm2+/p53– in well-differentiated/dedifferentiated liposarcomas (22). However, only a minimal percentage of RISHN presents with this combination, making the widespread possibility of using Mdm2 inhibitors very unlikely (21, 22).

## Clinical and radiological features

Zhu et al. summarized the features of 323 cases of RISHN in the literature, finding RIOS as the most common (34.1%) histotype, followed by fibrosarcoma (RIFS, 19.2%), undifferentiated pleomorphic sarcoma (RIUPS, 15.8%), previously named malignant fibrous histiocytoma, not otherwise specified sarcoma (10.7%), leiomyosarcoma (5.6%), and rhabdomyosarcoma (3.8%) (23). Other less common histological findings were malignant peripheral nerve sheath tumor, chondrosarcoma, angiosarcoma, carcinosarcoma, dermatofibrosarcoma, Kaposi's sarcoma, liposarcoma, low-grade myofibroblastic sarcoma, myofibroblastoma, and synovial sarcoma (23). This high variability was not reported in previous studies, in which RIUPS was the predominant histological subtype. This is probably due to recent advances in pathologic classification and diagnosis of sarcomas (24). For this reasons, the diagnosis of RIFS, even though still present in many retrospective studies, has been limited during the latest decades and divided into: fibromyxoid sarcoma, sclerosing epithelioid sarcoma, dermatofibrosarcoma protuberans, and fibrosarcomatous dermatofibrosarcoma protuberans (25).

The average latency between RT and RIS diagnosis is quite constant in most studies, even if the reported range can be extremely large (from <1 to 50 years) (23). Nonetheless, even though a minimum latency period of a few years has been routinely used to distinguish RISHN from *de novo* sarcomas, the majority of epidemiologic studies did not consider this time frame and also included early post-RT sarcomas.

From a clinical point of view, the early identification of RISHN may be difficult to make due to the induration and fibrosis of the irradiated field (26). The most common symptoms, essentially related to the site of occurrence of the tumor itself, are: asymmetry of the HN region, pain, trismus, epistaxis, diplopia, jaw numbness, and dysphagia, while sometimes RIS may be misdiagnosed as osteoradionecrosis (23, 26). Furthermore, the overall features of RISHN and *de novo* sarcomas make it impossible to define a clear distinction between these two entities: in fact, median age, gender ratio, median tumor size, and tumor grade are similar between RIS and osteoradionecrosis (27).

Radiological findings may be challenging due to the heterogeneous characteristics of RIS on CT and/or MR. Generally, RISHN may not be easily distinguished from primary cancer recurrence and/or second primary lesions but, in presence of a large size, rapidly growing, extensively invading, bony destructive lesion with highly heterogeneous appearance, and significant contrast enhancement, RISHN should be always suspected as the most probable entity (24).

On the other hand, RIOS is one of the RISHN that presents with sufficient differences to be distinguished from primary OS. In fact, the latter usually presents, in MR, with an intermediate T1-signal and marked T2-hyperintensity, while in RIOS T1-T2 findings are unpredictable and variable with a frequent presence of bony erosion (26). Moreover, the presence of an osteoid matrix does not correlate with the FDG uptake which in RIOS is frequently reduced, making such an interpretation much more complex (26). Finally, signals of RIOS are mainly solid and it may be confounded with more differentiated RI malignancies, such as meningioma (26). All these features are not coherent with *de novo* OS, and the variability of the related findings makes the spectrum of differential diagnoses wider.

## Treatment and prognosis

RIS are historically considered highly aggressive tumors, characterized by poor prognosis. Yeang et al. found an age more than 50, smoking history, tumor size, and grading to be significant negative prognosticators (27). However, most of the reports analyzed RISHN as if they were a single entity, even though, indeed, they include a wide range of possible histotypes, as mentioned above.

A recent study compared RIFS with its *de novo* counterpart: survival in patients treated for the former disease was significantly lower than that of the sporadic form (38.6 vs. 52.6% 5-year disease-specific survival, *p* = 0.0219). The authors suggested that this may be mainly determined by the impossibility to retreat patients with RT, one of the most important pillars of the therapeutic armamentarium (28). In fact, patients presenting these lesions less likely undergo further radiation treatment, while chemotherapy appears to be of limited benefit (28, 29).

The highly aggressive nature of RISHN, the frequent lack of feasibility of re-irradiation, and its limited chemosensitivity make surgery the most important treatment able to improve patient survival. Indeed, as for non-RIS, a significant increase in disease-specific and overall survivals has been shown in patients with macroscopically complete resection compared to cases in which incomplete surgery or re-irradiation alone had been performed (30). However, obtaining a resection with microscopically negative margins is not always possible, due to the nature of the lesion, RT-related sequelae, and the peculiarity of the HN anatomy. Moreover, during their growth RISHN do not respect fascial planes, and they often require wider and atypical resections (30). As a consequence, the surgical option is sometimes refused by the patient for the highly disfiguring and/or dysfunctional outcomes, while the aggressiveness necessary for an adequate resection may threaten vital or crucial structures, leading surgeons to seek for a compromise between quality of surgery and unbearable sequelae (30). For this reason, neoadjuvant therapy with re-irradiation (even though possible in <20% of cases) with or without chemotherapy (4, 27), when feasible, may be of help (31). This, indeed, underlies the absolute need for a comprehensive multidisciplinary approach in such a clinical scenario, in order to look for adjunctive tools in the management of a quite dismal disease.

## Conclusions

RISHN are rare and heterogeneous oncologic entities, with multiple atypical aspects in terms of occurrence, subtypes, clinical-radiological features, and therapeutic opportunities, which make the disease highly challenging to manage, stressing the need for evaluation by a multidisciplinary team in reference centers for treatment of sarcomas. IMRT/VMAT does not seem to increase the rate of post-RT sarcomas, even if further long-term studies are needed to validate the results found in the literature. Complete surgery remains the cornerstone of therapy. Genetic studies have not found any crucial mutations in RISHN, even though further investigations might be very helpful in finding possible new and effective drugs.

## Author contributions

LG: study design, data collection, and writing; FI: data collection and writing; MF, AG, SS, and CS: manuscript revision; CP: study design, data collection, writing, and manuscript revision.

### Conflict of interest statement

The authors declare that the research was conducted in the absence of any commercial or financial relationships that could be construed as a potential conflict of interest.
